# (4-Meth­oxy­phen­yl)(4-propyl­cyclo­hex­yl)methanone

**DOI:** 10.1107/S1600536813003644

**Published:** 2013-02-20

**Authors:** Ling Wang, Zi-Qian Chang, Jie Zhang, Cheng-Min Li

**Affiliations:** a Pharmacy Department of the Second Artillery General Hospital, Beijing 100088, People’s Republic of China

## Abstract

The asymmetric unit of the title compound, C_17_H_24_O_2_, contains two independent mol­ecules with different conformations. The least-squares plane through the cyclohexane ring makes dihedral angles of 52.9 (5) and 81.4 (4)° with the benzene ring in the two molecules. The cyclo­hexane ring adopts a chair conformation in both mol­ecules. In the crystal, weak C—H⋯O hydrogen bonds link mol­ecules related by translation in [100] into two crystallographically independent chains.

## Related literature

For the anti­hyperglycemic activity of SGLT2 inhibitors, see: Washburn (2009 ▶); Zhao *et al.* (2011 ▶); Shao *et al.* (2011 ▶). For the structure of (5-bromo-2-meth­oxy­phen­yl)(4-ethyl­cyclo­hex­yl)methanone, see: Wang *et al.* (2011 ▶).
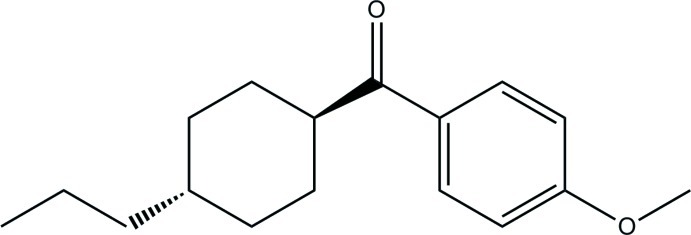


## Experimental

### 

#### Crystal data

C_17_H_24_O_2_
*M* *_r_* = 260.36
Triclinic, 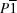

*a* = 5.679 (1) Å
*b* = 7.3260 (12) Å
*c* = 35.020 (4) Å
α = 93.74 (1)°
β = 91.877 (6)°
γ = 94.436 (1)°
*V* = 1448.5 (4) Å^3^
*Z* = 4
Mo *K*α radiation
μ = 0.08 mm^−1^
*T* = 113 K
0.18 × 0.16 × 0.14 mm

#### Data collection

Rigaku Saturn724 CCD diffractometer
Absorption correction: multi-scan (*CrystalClear*; Rigaku/MSC, 2009 ▶) *T*_min_ = 0.986, *T*_max_ = 0.989
14496 measured reflections
6762 independent reflections
5234 reflections with *I* > 2σ(*I*)
*R*_int_ = 0.027

#### Refinement

*R*[*F* ^2^ > 2σ(*F* ^2^)] = 0.045
*wR*(*F* ^2^) = 0.129
*S* = 1.03
6762 reflections
347 parameters
H-atom parameters constrained
Δρ_max_ = 0.37 e Å^−3^
Δρ_min_ = −0.21 e Å^−3^

### 

Data collection: *CrystalClear* (Rigaku/MSC, 2009 ▶); cell refinement: *CrystalClear*; data reduction: *CrystalClear*; program(s) used to solve structure: *SHELXS97* (Sheldrick, 2008 ▶); program(s) used to refine structure: *SHELXL97* (Sheldrick, 2008 ▶); molecular graphics: *SHELXTL* (Sheldrick, 2008 ▶); software used to prepare material for publication: *SHELXTL*.

## Supplementary Material

Crystal structure: contains datablock(s) I, global. DOI: 10.1107/S1600536813003644/cv5385sup1.cif

Structure factors: contains datablock(s) I. DOI: 10.1107/S1600536813003644/cv5385Isup2.hkl

Supplementary material file. DOI: 10.1107/S1600536813003644/cv5385Isup3.cml

Additional supplementary materials:  crystallographic information; 3D view; checkCIF report

## Figures and Tables

**Table 1:** Hydrogen-bond geometry (Å, °)

*D*—H⋯*A*	*D*—H	H⋯*A*	*D*⋯*A*	*D*—H⋯*A*
C9—H9⋯O1^i^	1.00	2.58	3.4661 (14)	147
C26—H26⋯O3^ii^	1.00	2.58	3.5200 (14)	156

Symmetry codes: (i) 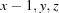; (ii) 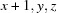.

**Fig. 1.**
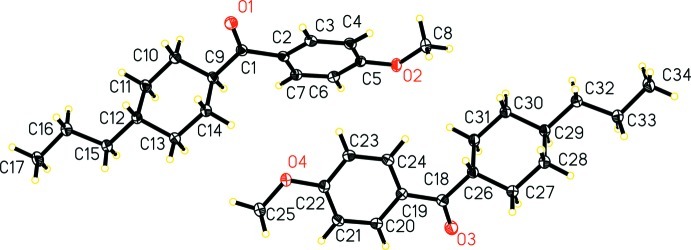
Two independent molecules of the title compound showing the atomic numbering and 40% probability displacement ellipsoids.

## supplementary crystallographic information

### Comment

SGLT2 inhibitors constitute new class of hyperglycemic agents, and the most advanced drug dapagliflozin has been approved recently in EU for the treatment of type 2 diabetes (Washburn, 2009). The title compound has been obtained in our laboratory as an intermediate used in the synthesis of SGLT2 inhibitors (Zhao *et al.*, 2011; Shao *et al.*, 2011).

The asymmetric unit of the title compound, C_17_H_24_O_2_, contains two independent molecules differing in conformations. In one independent molecule, the mean planes of C27/C28/C30/C31 and benzene ring C19–C24 form a dihedral angle of 52.9 (5)°, while in another independent molecule, the mean planes of C10/C11/C13/C14 and benzene ring C2–C7 form a dihedral angle of 81.4 (4)°. The cyclohexane ring adopts a chair conformation in both molecules. All bond lengths are normal and correspond to those observed in the related compound (Wang *et al.*, 2011).

In the crystal, weak intermolecular C—H···O hydrogen bonds (Table 1) link the molecules related by translation in [100] into two crystallographically independent chains.

### Experimental

17.03 g (0.1 mol) of *trans*-4-propylcyclohexanecarboxylic acid was stirred in 150 ml of dried dichloromethane at room temperature, followed by dropwise addition of 17.80 g (0.13 mol) of freshly distilled oxalyl chloride and 0.1 ml of dried DMF. The resulting mixture was stirred at room temperature for 5 h and evaporated in vacuo to remove the solvent and excessive oxalyl chloride to give a residue, which was dissolved in 100 ml of dried dichloromethane followed by addition of 10.81 g (0.1 mol) of anisole. The mixture thus obtained was stirred at 10 centigrade followed by addition of 14.67 g (0.11 mol) of AlCl_3_ portionwise. The reaction mixture was then stirred at room temperature overnight, poured into 300 ml of ice-water and extracted with 100 ml, three times of dichloromethane. The combined extracts were washed with brine, dried over Na2SO4 and evaporated to dryness. The residue was purified by column chromatography to afford the pure title compound as colorless crystals. The single crystals suitable for single-crystal X-ray diffraction were obtained by slow evaporation at room temperature of a 0.2 *M* solution of the title compound in dichloromethane/hexane (1/15).

### Refinement

All H atoms were geometrically positioned (C–H = 0.95–1.00 Å) , and included in the final cycles of refinement using a riding model, with *U*_iso_(H) = 1.2–1.5 *U*_eq_(C).

### Crystal data

**Table:**

C_17_H_24_O_2_	*Z* = 4
*M**_r_* = 260.36	*F*(000) = 568
Triclinic, *P*1	*D*_x_ = 1.194 Mg m^−^^3^
Hall symbol: -P 1	Mo *K*α radiation, λ = 0.71075 Å
*a* = 5.679 (1) Å	Cell parameters from 3880 reflections
*b* = 7.3260 (12) Å	θ = 1.7–27.9°
*c* = 35.020 (4) Å	µ = 0.08 mm^−^^1^
α = 93.74 (1)°	*T* = 113 K
β = 91.877 (6)°	Prism, colorless
γ = 94.436 (1)°	0.18 × 0.16 × 0.14 mm
*V* = 1448.5 (4) Å^3^	

### Data collection

**Table:**

Rigaku Saturn724 CCD diffractometer	6762 independent reflections
Radiation source: rotating anode	5234 reflections with *I* > 2σ(*I*)
Multilayer monochromator	*R*_int_ = 0.027
Detector resolution: 14.222 pixels mm^-1^	θ_max_ = 27.9°, θ_min_ = 1.8°
ω scans	*h* = −7→7
Absorption correction: multi-scan (*CrystalClear*; Rigaku/MSC, 2009)	*k* = −9→9
*T*_min_ = 0.986, *T*_max_ = 0.989	*l* = −45→45
14496 measured reflections	

### Refinement

**Table:**

Refinement on *F*^2^	Primary atom site location: structure-invariant direct methods
Least-squares matrix: full	Secondary atom site location: difference Fourier map
*R*[*F*^2^ > 2σ(*F*^2^)] = 0.045	Hydrogen site location: inferred from neighbouring sites
*wR*(*F*^2^) = 0.129	H-atom parameters constrained
*S* = 1.03	*w* = 1/[σ^2^(*F*_o_^2^) + (0.0748*P*)^2^] where *P* = (*F*_o_^2^ + 2*F*_c_^2^)/3
6762 reflections	(Δ/σ)_max_ = 0.001
347 parameters	Δρ_max_ = 0.37 e Å^−^^3^
0 restraints	Δρ_min_ = −0.21 e Å^−^^3^

### Special details

**Table:**

Geometry. All e.s.d.'s (except the e.s.d. in the dihedral angle between two l.s. planes) are estimated using the full covariance matrix. The cell e.s.d.'s are taken into account individually in the estimation of e.s.d.'s in distances, angles and torsion angles; correlations between e.s.d.'s in cell parameters are only used when they are defined by crystal symmetry. An approximate (isotropic) treatment of cell e.s.d.'s is used for estimating e.s.d.'s involving l.s. planes.
Refinement. Refinement of *F*^2^ against ALL reflections. The weighted *R*-factor *wR* and goodness of fit *S* are based on *F*^2^, conventional *R*-factors *R* are based on *F*, with *F* set to zero for negative *F*^2^. The threshold expression of *F*^2^ > σ(*F*^2^) is used only for calculating *R*-factors(gt) *etc*. and is not relevant to the choice of reflections for refinement. *R*-factors based on *F*^2^ are statistically about twice as large as those based on *F*, and *R*- factors based on ALL data will be even larger.

### Fractional atomic coordinates and isotropic or equivalent isotropic displacement parameters (Å^2^)

**Table:**

	*x*	*y*	*z*	*U*_iso_*/*U*_eq_	
O1	0.99880 (12)	0.35024 (11)	0.15912 (2)	0.0289 (2)	
O2	0.66126 (13)	0.59253 (10)	0.32334 (2)	0.02519 (18)	
O3	−0.01039 (13)	1.20793 (12)	0.33989 (2)	0.0331 (2)	
O4	0.30237 (13)	0.86232 (11)	0.18076 (2)	0.02688 (19)	
C1	0.79648 (17)	0.37013 (14)	0.16884 (3)	0.0199 (2)	
C2	0.75366 (17)	0.43668 (13)	0.20893 (3)	0.0182 (2)	
C3	0.94045 (17)	0.53011 (13)	0.23066 (3)	0.0200 (2)	
H3	1.0873	0.5551	0.2190	0.024*	
C4	0.91789 (17)	0.58768 (14)	0.26880 (3)	0.0210 (2)	
H4	1.0461	0.6539	0.2830	0.025*	
C5	0.70437 (18)	0.54689 (13)	0.28593 (3)	0.0197 (2)	
C6	0.51423 (17)	0.45635 (13)	0.26461 (3)	0.0207 (2)	
H6	0.3679	0.4310	0.2763	0.025*	
C7	0.53761 (17)	0.40306 (13)	0.22639 (3)	0.0200 (2)	
H7	0.4062	0.3432	0.2119	0.024*	
C8	0.8581 (2)	0.66708 (16)	0.34736 (3)	0.0295 (3)	
H8A	0.9838	0.5827	0.3462	0.035*	
H8B	0.8084	0.6839	0.3738	0.035*	
H8C	0.9171	0.7858	0.3386	0.035*	
C9	0.59203 (17)	0.33767 (14)	0.13965 (3)	0.0208 (2)	
H9	0.4431	0.3066	0.1531	0.025*	
C10	0.63021 (19)	0.18261 (14)	0.10969 (3)	0.0246 (2)	
H10A	0.7868	0.2058	0.0986	0.030*	
H10B	0.6292	0.0652	0.1222	0.030*	
C11	0.43893 (19)	0.16664 (14)	0.07775 (3)	0.0248 (2)	
H11A	0.2841	0.1324	0.0886	0.030*	
H11B	0.4724	0.0677	0.0585	0.030*	
C12	0.42516 (18)	0.34571 (14)	0.05803 (3)	0.0216 (2)	
H12	0.5817	0.3762	0.0468	0.026*	
C13	0.38275 (19)	0.49986 (15)	0.08810 (3)	0.0240 (2)	
H13A	0.3811	0.6171	0.0756	0.029*	
H13B	0.2260	0.4743	0.0990	0.029*	
C14	0.57195 (19)	0.51957 (14)	0.12039 (3)	0.0241 (2)	
H14A	0.5325	0.6157	0.1398	0.029*	
H14B	0.7263	0.5589	0.1100	0.029*	
C15	0.23631 (18)	0.33204 (15)	0.02564 (3)	0.0242 (2)	
H15A	0.0826	0.2907	0.0360	0.029*	
H15B	0.2219	0.4561	0.0166	0.029*	
C16	0.2837 (2)	0.20226 (16)	−0.00861 (3)	0.0273 (2)	
H16A	0.4423	0.2373	−0.0180	0.033*	
H16B	0.2845	0.0758	−0.0002	0.033*	
C17	0.1010 (2)	0.20485 (17)	−0.04126 (3)	0.0315 (3)	
H17A	−0.0554	0.1643	−0.0325	0.038*	
H17B	0.1419	0.1221	−0.0627	0.038*	
H17C	0.0987	0.3298	−0.0496	0.038*	
C18	0.18165 (18)	1.15443 (14)	0.33140 (3)	0.0213 (2)	
C19	0.21375 (17)	1.07129 (13)	0.29201 (3)	0.0192 (2)	
C20	0.03936 (17)	1.08960 (13)	0.26396 (3)	0.0200 (2)	
H20	−0.0977	1.1492	0.2707	0.024*	
C21	0.06192 (17)	1.02246 (14)	0.22640 (3)	0.0210 (2)	
H21	−0.0576	1.0371	0.2075	0.025*	
C22	0.26183 (18)	0.93344 (13)	0.21679 (3)	0.0209 (2)	
C23	0.43692 (17)	0.91194 (14)	0.24449 (3)	0.0212 (2)	
H23	0.5717	0.8492	0.2379	0.025*	
C24	0.41410 (17)	0.98210 (14)	0.28166 (3)	0.0207 (2)	
H24	0.5356	0.9698	0.3004	0.025*	
C25	0.1211 (2)	0.87346 (16)	0.15194 (3)	0.0308 (3)	
H25A	−0.0256	0.8085	0.1595	0.037*	
H25B	0.1695	0.8170	0.1276	0.037*	
H25C	0.0951	1.0025	0.1489	0.037*	
C26	0.38631 (17)	1.17056 (14)	0.36072 (3)	0.0203 (2)	
H26	0.5374	1.1922	0.3472	0.024*	
C27	0.36464 (18)	1.33026 (14)	0.39046 (3)	0.0230 (2)	
H27A	0.3720	1.4468	0.3776	0.028*	
H27B	0.2095	1.3151	0.4025	0.028*	
C28	0.56124 (18)	1.33969 (14)	0.42140 (3)	0.0240 (2)	
H28A	0.5397	1.4424	0.4404	0.029*	
H28B	0.7156	1.3647	0.4096	0.029*	
C29	0.56315 (18)	1.16106 (14)	0.44176 (3)	0.0220 (2)	
H29	0.4071	1.1399	0.4538	0.026*	
C30	0.58677 (19)	1.00186 (14)	0.41190 (3)	0.0235 (2)	
H30A	0.7421	1.0185	0.4000	0.028*	
H30B	0.5812	0.8853	0.4248	0.028*	
C31	0.39091 (18)	0.98958 (14)	0.38069 (3)	0.0231 (2)	
H31A	0.2364	0.9614	0.3922	0.028*	
H31B	0.4163	0.8884	0.3615	0.028*	
C32	0.75613 (19)	1.16748 (15)	0.47336 (3)	0.0242 (2)	
H32A	0.7643	1.0425	0.4823	0.029*	
H32B	0.9100	1.2033	0.4624	0.029*	
C33	0.7207 (2)	1.29943 (15)	0.50788 (3)	0.0268 (2)	
H33A	0.7267	1.4263	0.4996	0.032*	
H33B	0.5621	1.2702	0.5179	0.032*	
C34	0.9062 (2)	1.28984 (16)	0.53987 (3)	0.0307 (3)	
H34A	0.9018	1.1644	0.5481	0.037*	
H34B	0.8731	1.3743	0.5615	0.037*	
H34C	1.0631	1.3246	0.5305	0.037*	

### Atomic displacement parameters (Å^2^)

**Table:**

	*U*^11^	*U*^22^	*U*^33^	*U*^12^	*U*^13^	*U*^23^
O1	0.0202 (4)	0.0423 (5)	0.0243 (4)	0.0054 (3)	0.0018 (3)	−0.0008 (3)
O2	0.0282 (4)	0.0283 (4)	0.0184 (4)	0.0021 (3)	0.0006 (3)	−0.0026 (3)
O3	0.0219 (4)	0.0504 (5)	0.0271 (4)	0.0089 (4)	0.0020 (3)	−0.0033 (4)
O4	0.0308 (4)	0.0312 (4)	0.0187 (4)	0.0075 (3)	−0.0016 (3)	−0.0025 (3)
C1	0.0203 (5)	0.0204 (5)	0.0191 (5)	0.0023 (4)	0.0004 (4)	0.0026 (4)
C2	0.0188 (5)	0.0171 (5)	0.0190 (5)	0.0026 (4)	−0.0008 (4)	0.0035 (4)
C3	0.0182 (5)	0.0200 (5)	0.0221 (5)	0.0015 (4)	0.0001 (4)	0.0041 (4)
C4	0.0192 (5)	0.0196 (5)	0.0239 (5)	0.0007 (4)	−0.0028 (4)	0.0016 (4)
C5	0.0250 (5)	0.0171 (5)	0.0175 (5)	0.0047 (4)	0.0008 (4)	0.0024 (4)
C6	0.0195 (5)	0.0187 (5)	0.0243 (5)	0.0008 (4)	0.0037 (4)	0.0035 (4)
C7	0.0180 (5)	0.0176 (5)	0.0240 (5)	0.0000 (4)	−0.0010 (4)	0.0012 (4)
C8	0.0341 (6)	0.0328 (6)	0.0206 (5)	0.0039 (5)	−0.0051 (4)	−0.0045 (4)
C9	0.0184 (5)	0.0265 (5)	0.0174 (5)	0.0024 (4)	−0.0004 (4)	0.0007 (4)
C10	0.0285 (6)	0.0226 (5)	0.0226 (5)	0.0060 (4)	−0.0039 (4)	−0.0010 (4)
C11	0.0290 (6)	0.0229 (5)	0.0217 (5)	0.0019 (4)	−0.0047 (4)	−0.0021 (4)
C12	0.0217 (5)	0.0264 (5)	0.0166 (5)	0.0032 (4)	−0.0003 (4)	−0.0006 (4)
C13	0.0291 (6)	0.0250 (5)	0.0185 (5)	0.0082 (4)	−0.0028 (4)	−0.0001 (4)
C14	0.0282 (6)	0.0242 (5)	0.0198 (5)	0.0057 (4)	−0.0026 (4)	−0.0021 (4)
C15	0.0261 (6)	0.0284 (6)	0.0182 (5)	0.0055 (4)	−0.0022 (4)	−0.0005 (4)
C16	0.0319 (6)	0.0291 (6)	0.0207 (5)	0.0056 (5)	−0.0028 (4)	−0.0022 (4)
C17	0.0382 (7)	0.0336 (6)	0.0215 (5)	0.0033 (5)	−0.0072 (5)	−0.0029 (5)
C18	0.0203 (5)	0.0230 (5)	0.0208 (5)	0.0008 (4)	0.0020 (4)	0.0038 (4)
C19	0.0188 (5)	0.0179 (5)	0.0207 (5)	−0.0016 (4)	0.0005 (4)	0.0028 (4)
C20	0.0173 (5)	0.0191 (5)	0.0237 (5)	0.0001 (4)	0.0007 (4)	0.0034 (4)
C21	0.0205 (5)	0.0204 (5)	0.0221 (5)	0.0002 (4)	−0.0031 (4)	0.0037 (4)
C22	0.0252 (5)	0.0178 (5)	0.0196 (5)	−0.0002 (4)	0.0024 (4)	0.0019 (4)
C23	0.0204 (5)	0.0200 (5)	0.0238 (5)	0.0028 (4)	0.0020 (4)	0.0034 (4)
C24	0.0193 (5)	0.0216 (5)	0.0215 (5)	0.0008 (4)	−0.0009 (4)	0.0046 (4)
C25	0.0371 (7)	0.0338 (6)	0.0211 (5)	0.0078 (5)	−0.0053 (4)	−0.0025 (4)
C26	0.0197 (5)	0.0229 (5)	0.0181 (5)	0.0016 (4)	0.0009 (4)	0.0010 (4)
C27	0.0250 (5)	0.0231 (5)	0.0209 (5)	0.0045 (4)	−0.0003 (4)	0.0003 (4)
C28	0.0267 (6)	0.0231 (5)	0.0218 (5)	0.0023 (4)	−0.0017 (4)	−0.0018 (4)
C29	0.0229 (5)	0.0251 (5)	0.0181 (5)	0.0030 (4)	0.0003 (4)	−0.0001 (4)
C30	0.0288 (6)	0.0226 (5)	0.0192 (5)	0.0049 (4)	−0.0012 (4)	0.0001 (4)
C31	0.0260 (5)	0.0228 (5)	0.0202 (5)	0.0010 (4)	−0.0001 (4)	0.0001 (4)
C32	0.0259 (6)	0.0274 (6)	0.0196 (5)	0.0057 (4)	−0.0017 (4)	−0.0002 (4)
C33	0.0319 (6)	0.0275 (6)	0.0207 (5)	0.0051 (4)	−0.0020 (4)	−0.0017 (4)
C34	0.0361 (7)	0.0326 (6)	0.0226 (5)	0.0027 (5)	−0.0063 (5)	−0.0010 (4)

### Geometric parameters (Å, º)

**Table:**

O1—C1	1.2256 (12)	C17—H17A	0.9800
O2—C5	1.3657 (11)	C17—H17B	0.9800
O2—C8	1.4280 (12)	C17—H17C	0.9800
O3—C18	1.2261 (12)	C18—C19	1.4937 (13)
O4—C22	1.3669 (11)	C18—C26	1.5178 (14)
O4—C25	1.4284 (12)	C19—C20	1.3921 (13)
C1—C2	1.4897 (13)	C19—C24	1.4014 (14)
C1—C9	1.5157 (13)	C20—C21	1.3874 (13)
C2—C3	1.3927 (13)	C20—H20	0.9500
C2—C7	1.4007 (13)	C21—C22	1.3922 (14)
C3—C4	1.3872 (13)	C21—H21	0.9500
C3—H3	0.9500	C22—C23	1.3913 (14)
C4—C5	1.3909 (14)	C23—C24	1.3816 (13)
C4—H4	0.9500	C23—H23	0.9500
C5—C6	1.3910 (14)	C24—H24	0.9500
C6—C7	1.3834 (13)	C25—H25A	0.9800
C6—H6	0.9500	C25—H25B	0.9800
C7—H7	0.9500	C25—H25C	0.9800
C8—H8A	0.9800	C26—C27	1.5298 (13)
C8—H8B	0.9800	C26—C31	1.5404 (14)
C8—H8C	0.9800	C26—H26	1.0000
C9—C10	1.5285 (13)	C27—C28	1.5246 (14)
C9—C14	1.5426 (15)	C27—H27A	0.9900
C9—H9	1.0000	C27—H27B	0.9900
C10—C11	1.5259 (14)	C28—C29	1.5311 (15)
C10—H10A	0.9900	C28—H28A	0.9900
C10—H10B	0.9900	C28—H28B	0.9900
C11—C12	1.5278 (15)	C29—C32	1.5277 (14)
C11—H11A	0.9900	C29—C30	1.5316 (13)
C11—H11B	0.9900	C29—H29	1.0000
C12—C15	1.5284 (14)	C30—C31	1.5270 (14)
C12—C13	1.5314 (13)	C30—H30A	0.9900
C12—H12	1.0000	C30—H30B	0.9900
C13—C14	1.5262 (14)	C31—H31A	0.9900
C13—H13A	0.9900	C31—H31B	0.9900
C13—H13B	0.9900	C32—C33	1.5263 (13)
C14—H14A	0.9900	C32—H32A	0.9900
C14—H14B	0.9900	C32—H32B	0.9900
C15—C16	1.5253 (13)	C33—C34	1.5210 (15)
C15—H15A	0.9900	C33—H33A	0.9900
C15—H15B	0.9900	C33—H33B	0.9900
C16—C17	1.5207 (15)	C34—H34A	0.9800
C16—H16A	0.9900	C34—H34B	0.9800
C16—H16B	0.9900	C34—H34C	0.9800
			
C5—O2—C8	117.03 (8)	H17B—C17—H17C	109.5
C22—O4—C25	117.07 (8)	O3—C18—C19	119.74 (9)
O1—C1—C2	119.73 (9)	O3—C18—C26	120.49 (9)
O1—C1—C9	120.15 (8)	C19—C18—C26	119.77 (8)
C2—C1—C9	120.03 (8)	C20—C19—C24	118.60 (9)
C3—C2—C7	118.32 (9)	C20—C19—C18	118.07 (9)
C3—C2—C1	118.42 (9)	C24—C19—C18	123.28 (9)
C7—C2—C1	123.20 (8)	C21—C20—C19	121.24 (9)
C4—C3—C2	121.78 (9)	C21—C20—H20	119.4
C4—C3—H3	119.1	C19—C20—H20	119.4
C2—C3—H3	119.1	C20—C21—C22	119.19 (9)
C3—C4—C5	118.89 (9)	C20—C21—H21	120.4
C3—C4—H4	120.6	C22—C21—H21	120.4
C5—C4—H4	120.6	O4—C22—C23	115.43 (9)
O2—C5—C4	124.58 (9)	O4—C22—C21	124.12 (9)
O2—C5—C6	115.13 (9)	C23—C22—C21	120.46 (9)
C4—C5—C6	120.28 (9)	C24—C23—C22	119.76 (9)
C7—C6—C5	120.20 (9)	C24—C23—H23	120.1
C7—C6—H6	119.9	C22—C23—H23	120.1
C5—C6—H6	119.9	C23—C24—C19	120.73 (9)
C6—C7—C2	120.46 (9)	C23—C24—H24	119.6
C6—C7—H7	119.8	C19—C24—H24	119.6
C2—C7—H7	119.8	O4—C25—H25A	109.5
O2—C8—H8A	109.5	O4—C25—H25B	109.5
O2—C8—H8B	109.5	H25A—C25—H25B	109.5
H8A—C8—H8B	109.5	O4—C25—H25C	109.5
O2—C8—H8C	109.5	H25A—C25—H25C	109.5
H8A—C8—H8C	109.5	H25B—C25—H25C	109.5
H8B—C8—H8C	109.5	C18—C26—C27	111.17 (8)
C1—C9—C10	112.01 (8)	C18—C26—C31	109.14 (8)
C1—C9—C14	106.69 (8)	C27—C26—C31	109.91 (8)
C10—C9—C14	110.37 (8)	C18—C26—H26	108.9
C1—C9—H9	109.2	C27—C26—H26	108.9
C10—C9—H9	109.2	C31—C26—H26	108.9
C14—C9—H9	109.2	C28—C27—C26	111.38 (8)
C11—C10—C9	111.57 (8)	C28—C27—H27A	109.4
C11—C10—H10A	109.3	C26—C27—H27A	109.4
C9—C10—H10A	109.3	C28—C27—H27B	109.4
C11—C10—H10B	109.3	C26—C27—H27B	109.4
C9—C10—H10B	109.3	H27A—C27—H27B	108.0
H10A—C10—H10B	108.0	C27—C28—C29	111.88 (9)
C10—C11—C12	112.08 (9)	C27—C28—H28A	109.2
C10—C11—H11A	109.2	C29—C28—H28A	109.2
C12—C11—H11A	109.2	C27—C28—H28B	109.2
C10—C11—H11B	109.2	C29—C28—H28B	109.2
C12—C11—H11B	109.2	H28A—C28—H28B	107.9
H11A—C11—H11B	107.9	C32—C29—C28	113.07 (9)
C11—C12—C15	112.92 (9)	C32—C29—C30	111.21 (8)
C11—C12—C13	109.03 (8)	C28—C29—C30	108.89 (8)
C15—C12—C13	111.00 (8)	C32—C29—H29	107.8
C11—C12—H12	107.9	C28—C29—H29	107.8
C15—C12—H12	107.9	C30—C29—H29	107.8
C13—C12—H12	107.9	C31—C30—C29	112.01 (8)
C14—C13—C12	112.18 (8)	C31—C30—H30A	109.2
C14—C13—H13A	109.2	C29—C30—H30A	109.2
C12—C13—H13A	109.2	C31—C30—H30B	109.2
C14—C13—H13B	109.2	C29—C30—H30B	109.2
C12—C13—H13B	109.2	H30A—C30—H30B	107.9
H13A—C13—H13B	107.9	C30—C31—C26	111.24 (8)
C13—C14—C9	111.59 (9)	C30—C31—H31A	109.4
C13—C14—H14A	109.3	C26—C31—H31A	109.4
C9—C14—H14A	109.3	C30—C31—H31B	109.4
C13—C14—H14B	109.3	C26—C31—H31B	109.4
C9—C14—H14B	109.3	H31A—C31—H31B	108.0
H14A—C14—H14B	108.0	C33—C32—C29	114.64 (9)
C16—C15—C12	114.86 (9)	C33—C32—H32A	108.6
C16—C15—H15A	108.6	C29—C32—H32A	108.6
C12—C15—H15A	108.6	C33—C32—H32B	108.6
C16—C15—H15B	108.6	C29—C32—H32B	108.6
C12—C15—H15B	108.6	H32A—C32—H32B	107.6
H15A—C15—H15B	107.5	C34—C33—C32	112.69 (9)
C17—C16—C15	112.75 (9)	C34—C33—H33A	109.1
C17—C16—H16A	109.0	C32—C33—H33A	109.1
C15—C16—H16A	109.0	C34—C33—H33B	109.1
C17—C16—H16B	109.0	C32—C33—H33B	109.1
C15—C16—H16B	109.0	H33A—C33—H33B	107.8
H16A—C16—H16B	107.8	C33—C34—H34A	109.5
C16—C17—H17A	109.5	C33—C34—H34B	109.5
C16—C17—H17B	109.5	H34A—C34—H34B	109.5
H17A—C17—H17B	109.5	C33—C34—H34C	109.5
C16—C17—H17C	109.5	H34A—C34—H34C	109.5
H17A—C17—H17C	109.5	H34B—C34—H34C	109.5
			
O1—C1—C2—C3	20.66 (14)	O3—C18—C19—C20	12.36 (14)
C9—C1—C2—C3	−155.84 (9)	C26—C18—C19—C20	−168.16 (9)
O1—C1—C2—C7	−156.50 (10)	O3—C18—C19—C24	−170.06 (10)
C9—C1—C2—C7	26.99 (14)	C26—C18—C19—C24	9.42 (14)
C7—C2—C3—C4	0.78 (15)	C24—C19—C20—C21	−0.35 (14)
C1—C2—C3—C4	−176.52 (9)	C18—C19—C20—C21	177.34 (9)
C2—C3—C4—C5	1.54 (15)	C19—C20—C21—C22	0.70 (14)
C8—O2—C5—C4	−7.96 (14)	C25—O4—C22—C23	176.92 (9)
C8—O2—C5—C6	173.21 (9)	C25—O4—C22—C21	−3.15 (14)
C3—C4—C5—O2	178.66 (9)	C20—C21—C22—O4	−179.91 (9)
C3—C4—C5—C6	−2.56 (15)	C20—C21—C22—C23	0.02 (15)
O2—C5—C6—C7	−179.86 (9)	O4—C22—C23—C24	178.85 (9)
C4—C5—C6—C7	1.25 (15)	C21—C22—C23—C24	−1.08 (15)
C5—C6—C7—C2	1.13 (15)	C22—C23—C24—C19	1.44 (15)
C3—C2—C7—C6	−2.13 (15)	C20—C19—C24—C23	−0.73 (14)
C1—C2—C7—C6	175.04 (9)	C18—C19—C24—C23	−178.29 (9)
O1—C1—C9—C10	34.71 (13)	O3—C18—C26—C27	−27.44 (14)
C2—C1—C9—C10	−148.79 (9)	C19—C18—C26—C27	153.09 (9)
O1—C1—C9—C14	−86.17 (11)	O3—C18—C26—C31	93.96 (11)
C2—C1—C9—C14	90.32 (10)	C19—C18—C26—C31	−85.51 (11)
C1—C9—C10—C11	−172.99 (8)	C18—C26—C27—C28	176.42 (8)
C14—C9—C10—C11	−54.26 (11)	C31—C26—C27—C28	55.47 (11)
C9—C10—C11—C12	57.09 (12)	C26—C27—C28—C29	−57.59 (11)
C10—C11—C12—C15	179.38 (8)	C27—C28—C29—C32	−179.12 (8)
C10—C11—C12—C13	−56.75 (11)	C27—C28—C29—C30	56.73 (11)
C11—C12—C13—C14	56.27 (11)	C32—C29—C30—C31	178.27 (8)
C15—C12—C13—C14	−178.73 (8)	C28—C29—C30—C31	−56.49 (11)
C12—C13—C14—C9	−55.85 (12)	C29—C30—C31—C26	56.83 (11)
C1—C9—C14—C13	175.68 (8)	C18—C26—C31—C30	−177.24 (8)
C10—C9—C14—C13	53.76 (11)	C27—C26—C31—C30	−55.08 (11)
C11—C12—C15—C16	−66.01 (12)	C28—C29—C32—C33	67.56 (12)
C13—C12—C15—C16	171.21 (9)	C30—C29—C32—C33	−169.58 (9)
C12—C15—C16—C17	−175.54 (9)	C29—C32—C33—C34	175.41 (9)

### Hydrogen-bond geometry (Å, º)

**Table:**

*D*—H···*A*	*D*—H	H···*A*	*D*···*A*	*D*—H···*A*
C9—H9···O1^i^	1.00	2.58	3.4661 (14)	147
C26—H26···O3^ii^	1.00	2.58	3.5200 (14)	156

Symmetry codes: (i) *x*−1, *y*, *z*; (ii) *x*+1, *y*, *z*.

## References

[bb1] Rigaku/MSC (2009). *CrystalClear* Rigaku/MSC, The Woodlands, Texas, USA.

[bb2] Shao, H., Gao, Y. L., Lou, Y. Y., Wang, Y. L., Liu, W., Xu, W. R., Wang, J. W., Zhao, G. L. & Tang, L. D. (2011). *Chin. J. Org. Chem.* **31**, 836–842.

[bb3] Sheldrick, G. M. (2008). *Acta Cryst.* A**64**, 112–122. 10.1107/S0108767307043930 18156677

[bb4] Wang, L., Chang, Z., Ding, C., Shao, H. & Sun, J. (2011). *Acta Cryst.* E**67**, o1173. 10.1107/S1600536811013687 PMC3089268 21754478

[bb5] Washburn, W. N. (2009). *J. Med. Chem.* **52**, 1785–1794. 10.1021/jm8013019 19243175

[bb6] Zhao, W. J., Shi, Y. H., Zhao, G. L., Wang, Y. L., Shao, H., Tang, L. D. & Wang, J. W. (2011). *Chin. Chem. Lett.* **22**, 1215–1218.

